# Supplementary figures and images for: The association between HIV-related stigma, HIV knowledge and HIV late presenters among people living with HIV (PLHIV) attending public primary care clinic settings in Selangor
Source: PLoS One. 2024 Jul 22;19(7):e0306904. doi: 10.1371/journal.pone.0306904 (PMC11262653; doi:10.1371/journal.pone.0306904)

Supporting Documents

S1 Fig. Permission to use Berger HIV stigma scale questionnaire.


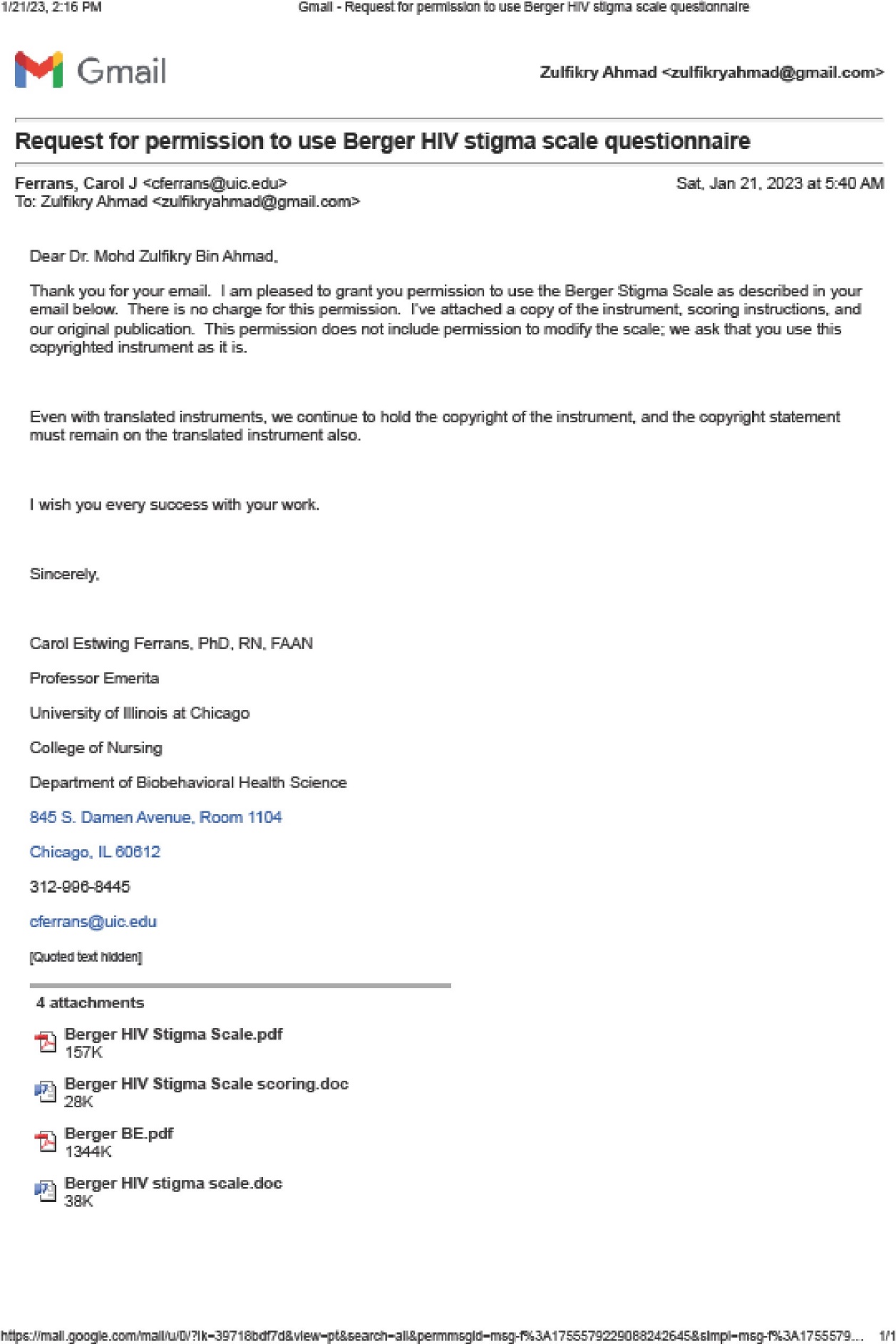

Supplement: S1 Fig — (DOCX) [file pone.0306904.s001.docx]

S2 Fig. Permission to use brief HIV-KQ-18 questionnaire.


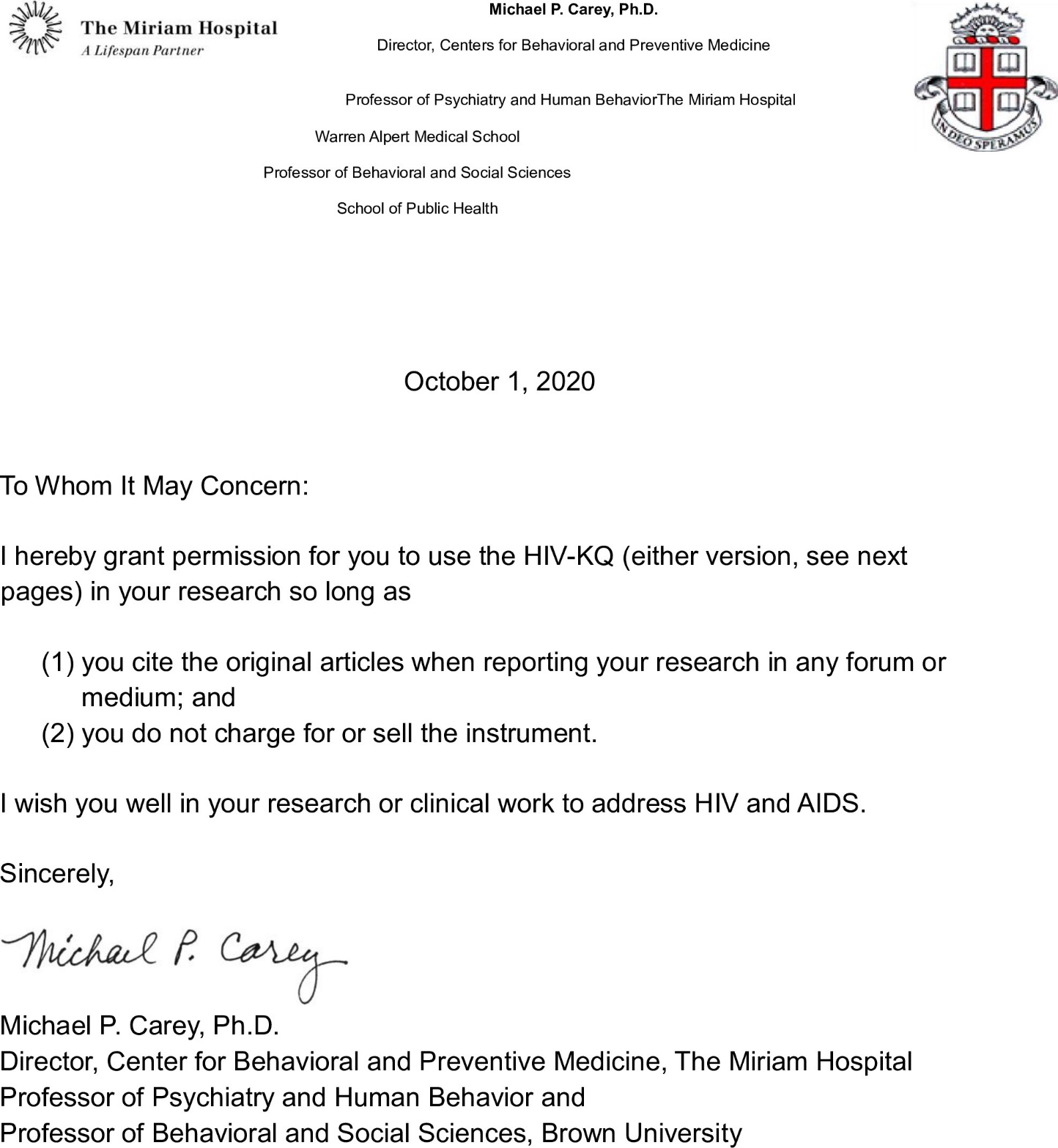

Supplement: S2 Fig — (DOCX) [file pone.0306904.s002.docx]

S4 Fig. Research Ethics Committee (REC) of Universiti Teknologi MARA Malaysia Approval.


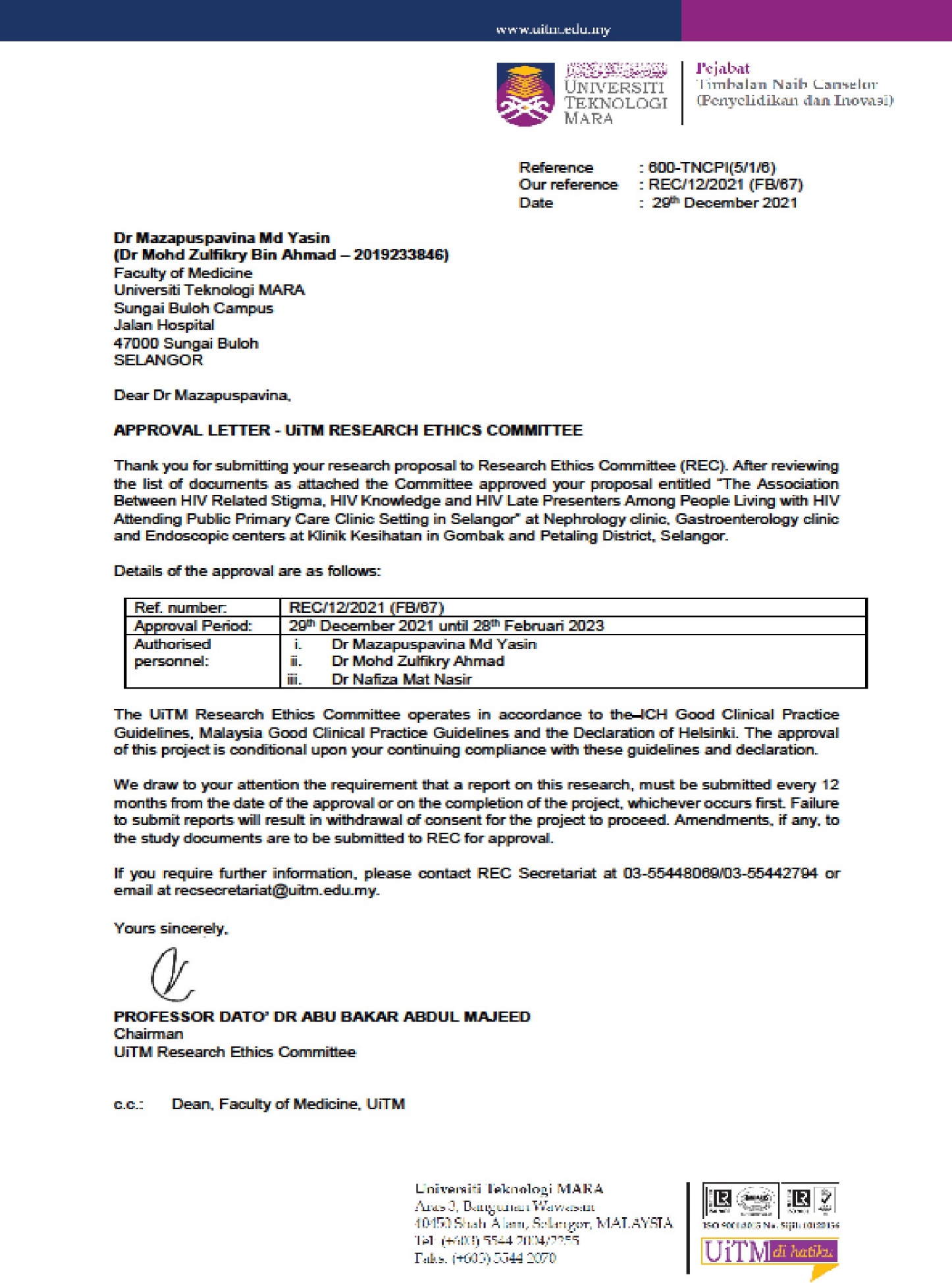

Supplement: S4 Fig — (DOCX) [file pone.0306904.s004.docx]
