# Supplementary material for: The association between HIV-related stigma, HIV knowledge and HIV late presenters among people living with HIV (PLHIV) attending public primary care clinic settings in Selangor
Source: PLoS One. 2024 Jul 22;19(7):e0306904. doi: 10.1371/journal.pone.0306904 (PMC11262653; doi:10.1371/journal.pone.0306904)

S3 Fig. Ethical approval the Medical Research and Ethics Committee (MREC) of the Ministry of Health Malaysia.


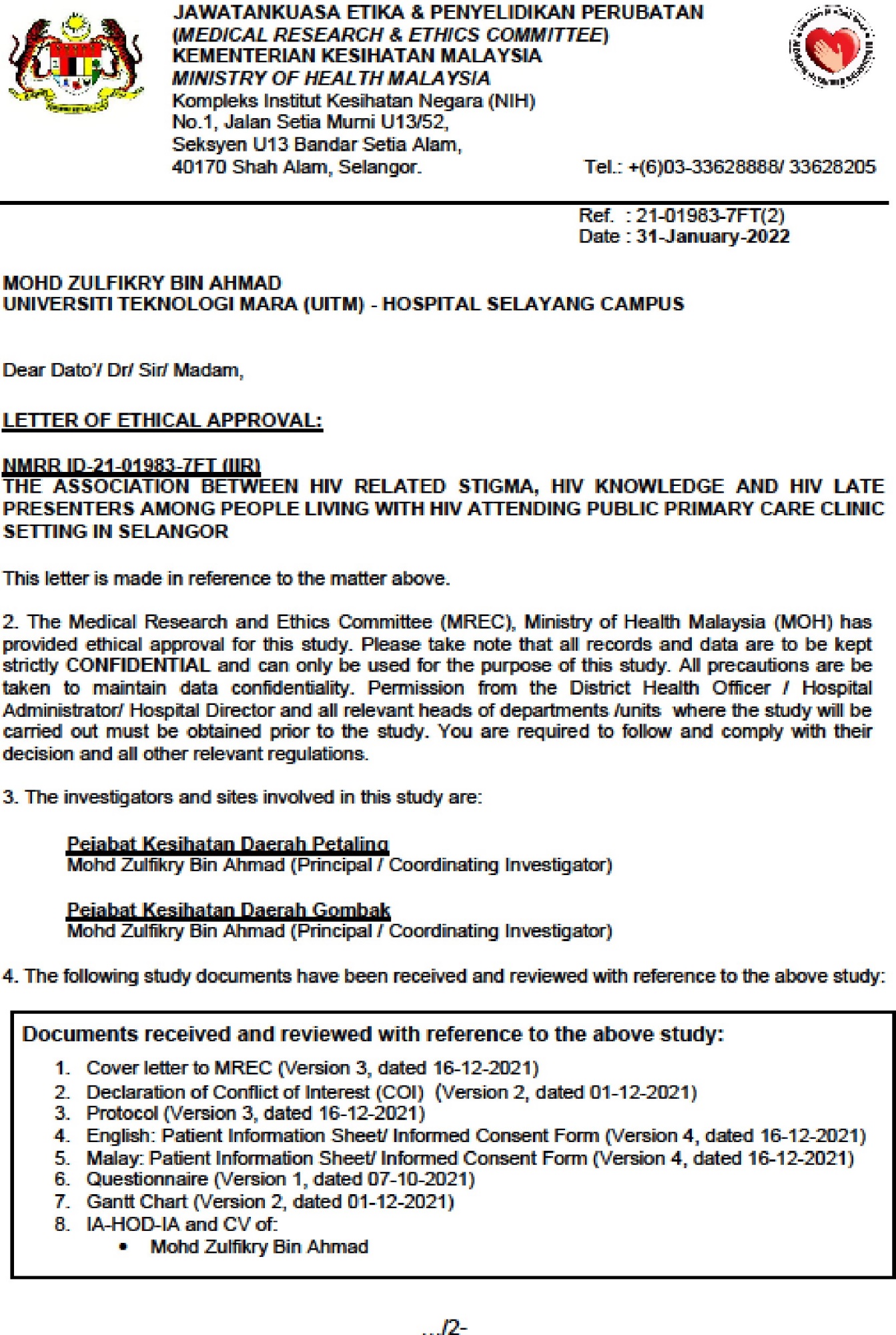

Supplement: S3 Fig — (DOCX) [file pone.0306904.s003.docx]
