# Supplementary material for: The association between HIV-related stigma, HIV knowledge and HIV late presenters among people living with HIV (PLHIV) attending public primary care clinic settings in Selangor
Source: PLoS One. 2024 Jul 22;19(7):e0306904. doi: 10.1371/journal.pone.0306904 (PMC11262653; doi:10.1371/journal.pone.0306904)
Supplement: Attachment — Submitted filename: Additional comments to the authors.docx [file pone.0306904.s005.docx]

Additional comments to the authors

Abstract

- The definition of late presenter should be placed right at the beginning or in the introduction section.
- The authors recommended “Effective intervention programs targeting early HIV diagnosis, improved HIV care, and treatment are necessary to address this issue” but didn’t think of factors that prevent early HIV diagnosis and treatment as they reported in the findings. Think about this and address it.

**INTRODUCTION**

- Please use the current 2022 UNAIDS data that are available online.
- Similarly, use current HIV data about Malaysia, 2021 data are nearly 2 years ago.
- It is a very long introduction which contains some unnecessary information. I would suggest deleting lines 54-67. This paragraph talks about the impact of HIV or late presentation for HIV care, while the focus of this study is on investigating factors associated with late presentation for HIV care.
- My biggest concern about the introduction section is that the synthesis of the existing findings from previous studies on factors associated with or influencing the presentation or access of PLHIV to HIV care or treatment is poorly done (see lines 68-95). There have been massive studies on this aspect globally and in Asia, which the authors need to consult and provide a good synthesis. Some resources are recommended below.
- The synthesis will help the authors to finally formulate the knowledge gap they wanted to bridge through this study or to state the novelty of their study and why it is important. This will become your contribution to knowledge.
- The authors can consult and use the following resources, which report on HIV stigma, HIV knowledge and various factors that influence access to HIV testing, care and treatment among PLHIV:
  - HIV Stigma and Moral Judgement: Qualitative Exploration of the Experiences of HIV Stigma and Discrimination among Married Men Living with HIV in Yogyakarta. Int. J. Environ. Res. Public Health **2020**, 17, 636. <https://doi.org/10.3390/ijerph17020636> <https://www.mdpi.com/1660-4601/17/2/636>
  - Stigma and Discrimination towards People Living with HIV in the Context of Families, Communities, and Healthcare Settings: A Qualitative Study in Indonesia. Int. J. Environ. Res. Public Health **2021**, 18, 5424. <https://doi.org/10.3390/ijerph18105424> <https://www.mdpi.com/1660-4601/18/10/5424>
  - [HIV stigma and discrimination: Perspectives and personal experiences of healthcare providers in Yogyakarta and Belu](https://scholar.google.com.au/citations?view_op=view_citation&hl=en&user=K7SUZ1YAAAAJ&citation_for_view=K7SUZ1YAAAAJ:JV2RwH3_ST0C). Frontiers in Medicine 8, 625787. <https://www.frontiersin.org/articles/10.3389/fmed.2021.625787/full>
  - Barriers to HIV testing among male clients of female sex workers in Indonesia. *Int J Equity Health* **17**, 68 (2018). <https://doi.org/10.1186/s12939-018-0782-4> <https://equityhealthj.biomedcentral.com/articles/10.1186/s12939-018-0782-4#citeas>
  - The Intention of Men Who Have Sex With Men to Participate in Voluntary Counseling and HIV Testing and Access Free Condoms in Indonesia. *American Journal of Men’s Health*. 2018;12(5):1175-1184. doi:[10.1177/1557988318779737](https://doi.org/10.1177/1557988318779737) <https://journals.sagepub.com/doi/full/10.1177/1557988318779737>
  - Barriers to Accessing HIV Care Services in Host Low and Middle Income Countries: Views and Experiences of Indonesian Male Ex-Migrant Workers Living with HIV. Int. J. Environ. Res. Public Health **2022**, 19, 14377. <https://doi.org/10.3390/ijerph192114377> <https://www.mdpi.com/1660-4601/19/21/14377>
  - Traditional Human Immunodeficiency Virus treatment and family and social influence as barriers to accessing HIV care services in Belu, Indonesia. PLoS ONE 17(7): e0264462. <https://doi.org/10.1371/journal.pone.0264462> <https://journals.plos.org/plosone/article?id=10.1371/journal.pone.0264462>

**DISCUSSION**

The authors stated: “To the best of our knowledge, this is the first study in Malaysia to determine the prevalence and explore the risk factors for HIV late presenters”. I do encourage the authors to consult the existing literature on this topic in Malaysia and globally and discuss your findings in light of previous findings, theories, etc. Please consult the following resources:

- Delayed HIV testing and treatment seeking, and associated support needs among people living with HIV in Malaysia: a qualitative study. <https://www.proquest.com/openview/bf841f3f539304a03b795ca784414a89/1?pq-origsite=gscholar&cbl=2037399>
- Factors Affecting Voluntary HIV Testing Among General Adult Population: A Cross-Sectional Study in Sarawak, Malaysia. <https://www.ncbi.nlm.nih.gov/pmc/articles/PMC7428414/>
- Late Presentation into Care of HIV Disease and Its Associated Factors in Asia: Results of TAHOD. <https://www.ncbi.nlm.nih.gov/pmc/articles/PMC4779961/>
- HIV Testing and awareness of HIV status among people who inject drugs in greater Kuala Lumpur, Malaysia. <https://www.tandfonline.com/doi/abs/10.1080/09540121.2017.1363852>
- Drivers of HIV self-testing among female sex workers: Findings from a multi-state study in Malaysia. <https://www.frontiersin.org/articles/10.3389/fmed.2023.1022746/full>

Additional resources:

- HIV/AIDS late presentation and its associated factors in China from 2010 to 2020: a systematic review and meta-analysis. *AIDS Res Ther* **18**, 96 (2021). <https://doi.org/10.1186/s12981-021-00415-2> <https://aidsrestherapy.biomedcentral.com/articles/10.1186/s12981-021-00415-2#citeas>
- Late presentation of HIV positive adults and its predictors to HIV/AIDS care in Ethiopia: a systematic review and meta-analysis. *BMC Infect Dis* **19**, 534 (2019). <https://doi.org/10.1186/s12879-019-4156-3> <https://bmcinfectdis.biomedcentral.com/articles/10.1186/s12879-019-4156-3#citeas>
