# Supplementary material for: The association between HIV-related stigma, HIV knowledge and HIV late presenters among people living with HIV (PLHIV) attending public primary care clinic settings in Selangor
Source: PLoS One. 2024 Jul 22;19(7):e0306904. doi: 10.1371/journal.pone.0306904 (PMC11262653; doi:10.1371/journal.pone.0306904)
Supplement: Attachment — Submitted filename: Response to reviewers.docx [file pone.0306904.s006.docx]

| **No** | **Comments** | **Action** |
| --- | --- | --- |
| 1. | Please ensure that your manuscript meets PLOS ONE's style requirements, including those for file naming. | Thank you for your comment. We have ensured that our manuscript meets PLOS ONE’s style requirements, including the naming. |
| 2. | Please note that funding information should not appear in any section or other areas of your manuscript. We will only publish funding information present in the Funding Statement section of the online submission form. Please remove any funding-related text from the manuscript. | We have removed the funding statement from the manuscript. |
| 3. | Please state what role the funders took in the study.  If the funders had no role, please state: "The funders had no role in study design, data collection and analysis, decision to publish, or preparation of the manuscript." | The funders had no role in study design, data collection and analysis, decision to publish, or preparation of the manuscript. |
| 4. | Your ethics statement should only appear in the Methods section of your manuscript. If your ethics statement is written in any section besides the Methods, please move it to the Methods section and delete it from any other section. | The ethics statement has been moved to the Methods section, and it has been deleted from any other section. |
| 5. | Please include captions for your Supporting Information files at the end of your manuscript, and update any in-text citations to match accordingly. Please see our Supporting Information guidelines for more information: | We have included supporting information captions at the end of the manuscript. |
| 6. | Abstract   - The definition of late presenter should be placed right at the beginning or in the introduction section. | We have moved the definition of late presenter at the beginning of the introduction section (Line 23-25). |
| 7. | - The authors recommended “Effective intervention programs targeting early HIV diagnosis, improved HIV care, and treatment are necessary to address this issue” but didn’t think of factors that prevent early HIV diagnosis and treatment as they reported in the findings. Think about this and address it. | We have revised our statement as follow to include our study findings: Interventions targeting stigma reduction and HIV education can promote early testing and prompt access to care, improving health outcomes for PLHIV. (Line 43-45) |
| 8. | **INTRODUCTION**   - Please use the current 2022 UNAIDS data that are available online. | We have used the current 2022 UNAIDS data with updated reference. |
| 9. | - Similarly, use current HIV data about Malaysia, 2021 data are nearly 2 years ago. | We have used the latest 2022 data published by Ministry of Health, Malaysia. |
| 10. | - It is a very long introduction which contains some unnecessary information. I would suggest deleting lines 54-67. This paragraph talks about the impact of HIV or late presentation for HIV care, while the focus of this study is on investigating factors associated with late presentation for HIV care. | We have deleted unnecessary information and summarized the impact of HIV late presentation as follow: (Line 73-77)  Late HIV diagnosis has significant adverse outcomes for individuals, including physical and psychological impacts, while also increasing the risk of onward transmission in society. In Malaysia, the financial burden of HIV-related expenses falls predominantly on domestic public funding, covering more than 80% of total expenditure in 2021, approximately RM82.9 million |
| a. | - My biggest concern about the introduction section is that the synthesis of the existing findings from previous studies on factors associated with or influencing the presentation or access of PLHIV to HIV care or treatment is poorly done (see lines 68-95). There have been massive studies on this aspect globally and in Asia, which the authors need to consult and provide a good synthesis. Some resources are recommended below. - The synthesis will help the authors to finally formulate the knowledge gap they wanted to bridge through this study or to state the novelty of their study and why it is important. This will become your contribution to knowledge. | From the original manuscript, lines 68-71 is focusing on factors associated with HIV late presentation. In the corrected manuscript, this information was retained at line 77-79.  Line 72-95 in the original manuscript focused on PLHIV only due to limited evidence on HIV late presentation related to stigma & knowledge. Therefore, based on the reviewer comment, we have incorporated 4 out 7 resources recommended to create a better synthesis. i.e more relevant risk factors to PLHIV (line 80-84) and stigma and discrimination (line 85-99). |
| 11. | **DISCUSSION**  The authors stated: “To the best of our knowledge, this is the first study in Malaysia to determine the prevalence and explore the risk factors for HIV late presenters”. I do encourage the authors to consult the existing literature on this topic in Malaysia and globally and discuss your findings in light of previous findings, theories, etc. | We have deleted the sentence “To the best of our knowledge, this is the first study in Malaysia to determine the prevalence and explore the risk factors for HIV late presenters” to avoid any misleading statement. We have incorporated suggested resources as part of our findings discussion. |
| 12. | About multivariate modeling. The author states that the modeling of multivariate analysis is based on the diagnosis of multicollinearity and a model fit examination (Hosmer-Lemeshow test). However, the differences between the variables in the univariate model and multivariate model in Table 4 are not clearly explained. Based on the results of the logistic regression analysis, my guess is that non-significant variables were not included in the multivariate analysis model. | To determine the association of HIV late presentation, we used simple logistic regression analysis to screen the independent variables. Those with P value <0.25 were selected to be further analyzed in the multiple logistic regression (meaning 0.25 or more were not included in our multivariable analysis). The multicollinearity and model fitness analysis were done to the final model to ensure that our model would not have any problem of multicollinearity and unfit model. We have made amendment and correction in the Data Analysis section (line 240-255). |
| 13. | Since multivariate adjustment is based on modeling and not on statistical probability and significance, I recommend avoiding the above method. Also, variables that are likely to have multicollinearity should be carefully considered and adjusted, rather than just being deleted.  First, the authors should clearly state the results of the diagnosis of collinearity. At least VIFs greater than or equal to 10 should be removed from the multivariate model. Therefore, authors need to be clear about which variables result in high VIF. | The process of checking multicollinearity and interaction problem were stated in the Data Analysis section (line 249-255). |
| 14. | After that, please consider the following. For example, there seems to be a high correlation between gender and marital status and MSM. There also seems to be a high correlation between Nationality and Ethnicity. The same goes for income, occupation, and education. These relationships can be easily inferred theoretically. As mentioned above, I strongly recommend that you select the collinearity diagnostic results and the variables that should be adjusted content-wise and theoretically. | Variables with a p-value <0.25 in simple logistic regression were age, ethnicity, marital status, education level, monthly income, level of HIV-related stigma, and level of HIV knowledge were entered to be analysed in MLogR. They were controlled as confounding factors by statistical method that was MLogR. Unfortunately, the nationality and occupation variables were not selected in this analysis because of poor significant value (P value >0.25). |
| 15. | The year of diagnosis or the number of years since diagnosis should be used as an adjustment variable. This study is a cross-sectional study, and we do not know whether current knowledge and stigma arose after or before diagnosis. Since you should at least know the year of diagnosis, we strongly recommend adjusting the year of diagnosis (or years since diagnosis). | This will be limitation of our study design as we could not determine whether current knowledge and stigma arose before or after the diagnosis. |
| 16. | Please indicate AIC, Hosmer-Lemeshow test, model chi-square value, and chi-square test results in the results section as model fit indicators for multivariate analysis results. | Hosmer-Lemshow test P value of 0.407 was added below the table 4. |
| 17. | Abstract: - Results: the authors provided the prevalence of late presenters (60%) with a confidence interval (CI) ranging from 55 to 65. As this is a prevalence, I cannot figure out how the authors derive the values of CI. Is it an aggregated value of the prevalence from many study settings so that they have the CI values? (the same thing later in the result section, while in the table 1 it was only the prevalence (n and %)) | The CI values were derived from one sample t-test analysis in the SPSS. Prevalence with a confidence interval value provide a more comprehensive picture of the data as it conveys the precision of estimate. |
| 18. | - Results: please re-check the results from the “tertiary education background” as I am not quite sure with the values provided there (aOR or the CI). Need to check the raw results from the software. | We have re-check the result from the software which showed similar outcome. |
| 19. | First sentence (line 31-32) need a reference | The first sentence citation is similar with the second sentence (line 50-53). |
| 20. | Stigma is not always imposed by outside parties. Internal stigma can also take place. The authors must provide information regarding this and the area in which they are concentrating their research. | We have added information regarding stigma as follow(Line 86-93): Stigma can be viewed from two perspectives, first is stigma experienced by PLHIV and second is stigma exhibited by the general population towards PLHIV. Our study is focusing on stigma perceived by PLHIV which can be categorized into enacted, anticipated and internalized stigma. Anticipated stigma was encountered by PLHIV due to prevailing HIV-related discrimination and stigma experienced by other PLHIV. Internalized stigma was influenced by individual moral judgements that linked HIV status with morally questionable behaviors, as well as their own negative self-assessment |
| 21. | Methods: - Line 116-117: “Multiple sample sizes were calculated based on different prevalence…” Please elaborate more this sentence, what did they mean by “different prevalence”? (from previously published studies?) | The different prevalence is based on independent variables in this study which include prevalence of HIV late presenters, level of HIV related stigma and level of HIV knowledge. We selected the sample size based on each independent variable, choosing the highest calculated sample size for this study. |
| 22. | Why “2019 onward”? Is it because of the country progress report 2019 or any other reason? | We want to include the recently diagnosed PLHIV to reduce reporting bias on level of HIV-related stigma and HIV knowledge. However considering COVID-19 pandemic, we decided to include those diagnosed since 2019 (one year before COVID-19) as recently diagnosed PLHIV. |
| 23. | Table 1: check the values of the percentages of some variables (not a 100% in total), i.e. males and females in the non-late presenters | The discrepancies because in males the value was 96.25 and females was 3.75. Both values were round up to single decimal point to standardize with all other variables. |
| 24. | Table 4: re-check the values of ORs and Cis (some of the values are weird) | We have rechecked the values of ORs and CIs in table 4, and the results remained the same. |
| 25. | Until the end of the results I could not find the CI values for the prevalence as they mentioned in the abstract, results (narrative), and discussion | The CI values for the prevalence was derived from one sample t-test analysis in the SPSS as mentioned previously. |
| 26. | Discussion: - - It is preferable to use fewer numbers in the discussion (numbers have been presented in the results). | We have reduced numbers in the discussion, for example in line 375-376:  The mean score of HIV-related stigma among all participants is consistent with the results of a previous local study conducted by Abdullah, A. (2020). |
