# Supplementary material for: The association between HIV-related stigma, HIV knowledge and HIV late presenters among people living with HIV (PLHIV) attending public primary care clinic settings in Selangor
Source: PLoS One. 2024 Jul 22;19(7):e0306904. doi: 10.1371/journal.pone.0306904 (PMC11262653; doi:10.1371/journal.pone.0306904)
Supplement: Attachment — Submitted filename: Response to reviewers_v2.docx [file pone.0306904.s007.docx]

| **No** | **Comments** | **Action** |
| --- | --- | --- |
| 1. | Please be more specific and clear about your research objective, L121~123. "...including HIV-related stigma and knowledge", but isn't this study focusing on both of these as independent variables? | The sentence has been rephrased to make the objective more specific and clear: “This research aims to determine the prevalence of HIV late presenters, and its association with HIV-related stigma, knowledge, and sociodemographic factors, among PLHIV who attend public primary care clinics in Selangor.” (Line 120-122) |
| 2. | Please explain how missing values are handled during analysis, along with its validity. | During data collection, we ensured the completeness of the filled questionnaires, resulting in no missing values for our analysis. |
| 3. | L250, The author sets the VIF standard at 10. Please provide the basis for that. | The variance inflation factor is a measure to analyze the magnitude of multicollinearity of model terms. A VIF less than 5 indicates a low correlation of that predictor with other predictors. A value between 5 and 10 indicates a moderate correlation, while VIF values larger than 10 are a sign for high, not tolerable correlation of model predictors (James et al. 2013).  The reference was added in the manuscript (Line 251).  James, G., Witten, D., Hastie, T., and Tibshirani, R. (eds.). (2013). An introduction to statistical learning: with applications in R. New York: Springer |
| 4. | About modeling in logistic regression analysis. It is theoretically insufficient to omit multivariate adjustment on the basis that the relationship between the adjustment variable and the dependent variable is not statistically significant in univariate analysis.  Since multicollinearity has already been taken into account, it is necessary to insert adjustment variables into the model and actively adjust the results, regardless of statistical significance, and present the results as the final model. | We agree with the reviewer that certain clinically important variables, though not statistically significant, may still be included in the MLogR model. We have included theese variables with p value <0.25, along with other clinically important factors such as age, gender, ethnicity, nationality, occupation, and monthly income, and mode of transmission. Despite this inclusion, the results remain unchanged. Additionally, the final model resulted in four predicted variables, making it a parsimonious model (simple and easy to explain model). (Line 244-245) |
